# Associations of *GHR, IGF-1* and *IGFBP-3* expression in adipose tissue cells with obesity-related alterations in corresponding circulating levels and adipose tissue function in children

**DOI:** 10.1080/21623945.2022.2148886

**Published:** 2022-11-22

**Authors:** Elena Kempf, Kathrin Landgraf, Tim Vogel, Ulrike Spielau, Robert Stein, Matthias Raschpichler, Jürgen Kratzsch, Wieland Kiess, Juraj Stanik, Antje Körner

**Affiliations:** a University of Leipzig, Medical Faculty, University Hospital for Children and Adolescents, Center for Pediatric Research, Leipzig, Germany; b Helmholtz Institute for Metabolic, Obesity and Vascular Research (HI-MAG) of the Helmholtz Zentrum München at the University of Leipzig and University Hospital Leipzig, Leipzig, Germany; c Medical Faculty, Department of Paediatric Radiology, University of Leipzig, Leipzig, Germany; d University of Leipzig, Medical Faculty, Institute of Laboratory Medicine, Clinical Chemistry and Molecular Diagnostics, Leipzig, Germany; e University of Leipzig, Medical Faculty, LIFE–Leipzig Research Center for Civilization Diseases, Leipzig, Germany; f Comenius University, Medical Faculty and National Institute of Children’s Diseases, Department of Pediatrics, Limbova 1, 83340 Bratislava, Slovakia, and Slovak Academy of Sciences, Biomedical Research Center, Institute of Experimental Endocrinology, DIABGENE Laboratory, Bratislava, Slovakia

**Keywords:** Obesity, children, adipose tissue, liver fat, growth hormone, childhood obesity, GHR, GHBP, IGF-1, IGFBP-3

## Abstract

Components of the growth hormone (GH) axis, such as insulin-like growth factor-1 (IGF-1), IGF-1 binding protein-3 (IGFBP-3), GH receptor (GHR) and GH-binding protein (GHBP), regulate growth and metabolic pathways. Here, we asked if serum levels of these factors are altered with overweight/obesity and if this is related to adipose tissue (AT) expression and/or increased fat mass. Furthermore, we hypothesized that expression of *GHR, IGF-1* and *IGFBP-3* is associated with AT function. Serum GHBP levels were increased in children with overweight/obesity throughout childhood, while for IGF-1 levels and the IGF-1/IGFBP-3 molar ratio obesity-related elevations were detectable until early puberty. Circulating levels did not correlate with AT expression of these factors, which was decreased with overweight/obesity. Independent from obesity, expression of *GHR, IGF-1* and *IGFBP-3* was related to AT dysfunction,and increased insulin levels. Serum GHBP was associated with liver fat percentage and transaminase levels. We conclude that obesity-related elevations in serum GHBP and IGF-1 are unlikely to be caused by increased AT mass and elevations in GHBP are more closely related to liver status in children. The diminished AT expression of these factors with childhood obesity may contribute to early AT dysfunction and a deterioration of the metabolic state.

## Introduction

Childhood obesity manifests early in life and may hence affect growth and sexual maturation [1]. Recently, we have shown that children with obesity have distinct growth dynamics compared to normal-weight children and that simultaneously serum levels of growth-related factors are altered with obesity [2]. Growth is a tightly regulated process, and the growth hormone (GH) axis is the major endocrine regulator of growth. The insulin-like growth factor-1 (IGF-1) is the key regulator of postnatal longitudinal growth, and its secretion is regulated by multiple players of the GH/IGF-1 axis [3]. Hypothalamic GH releasing hormone and somatostatin drive the pulsatile GH secretion from the anterior pituitary gland [4]. About 50% of GH is bound to GH binding protein (GHBP), which is the soluble form of the GH receptor (GHR) and regulates the half-life and bioavailability of GH [5]. In humans, GHBP is shedded from the cell surface by the tumour necrosis factor-α-converting enzyme [6], while in mice, GHBP is generated from an alternative splice variant [5] highlighting the limited comparability of regulation within the GH/IGF-1 axis in mouse and man and implying the importance of human studies. When GH binds to transmembrane GHR, expression of *IGF-1* and IGF-1 binding proteins (*IGFBPs*) [7–9] in the liver, but also in several other tissues [10], is induced. Predominantly, IGFBP-3 in complex with acid-labile subunit modulates the half-life and bioavailability of circulating IGF-1 [9]. By activating IGF-1 receptor (IGF-1R), IGF-1 finally induces cell proliferation and other metabolic effects in target tissues [11].

The liver is considered to be the main source of circulating GHBP [12,13], IGF-1 [14,15] and IGFBP-3 [16]. Nevertheless, the factors are additionally expressed in various other cell types, including adipose tissue (AT) cells [17–19], and there is still uncertainty about the contribution of other tissues to circulating levels [20,21]. Most of the studies addressing this question have been performed in mice and data regarding humans and specifically children, where the growth hormone axis is most important, is sparse.

The endocrine function of AT is disrupted with obesity [22]. Hence, obesity-associated changes in gene expression of *GHR, IGF-1* or *IGFBP-3* in AT and/or excess AT mass may contribute to alterations in circulating levels of these growth-related factors, however so far, there is no study investigating the associations between gene expression in AT cells and serum levels of these factors in children. Furthermore, AT expression may not only affect serum levels and growth in children but might also modulate AT function and hence contribute to the development of obesity-related comorbidities in children. GHR signalling for example is known to regulate triglyceride accumulation in adipocytes [23], while IGF-1 [24,25] and IGFBP-3 [9] have been suggested to be involved in AT expansion and adipogenic differentiation.

We hypothesized that circulating levels of GHBP, IGF-1 and IGFBP-3 are altered in children with overweight or obesity (overweight/obesity) compared to lean children and that gene expression of these factors in AT cells, also considering an increased fat mass with obesity, is related to those changes.

Furthermore, we assessed if gene expression of *GHR, IGF-1* and *IGFBP-3* in AT cells is related to overweight/obesity and AT function.

## Results

### Characteristics of the cohorts

All together, 306 children of the Leipzig AT Childhood Cohort (N = 209) and the Leipzig Atherobesity Childhood Cohort (N = 97) were included in this study. Anthropometric, laboratory and gene expression data as well as parameters for AT were compared between lean children and children with overweight/obesity (S1 Table and S2 Table). There was no significant difference in the distribution of sex, but children with overweight/obesity were slightly older, taller and more advanced in puberty than their lean peers. When stratified for pubertal stage, children with overweight/obesity were taller than their lean peers, whereas age was not different between the two weight groups from pubertal stage 2 onwards, pointing towards an obesity-associated gain in height (S1 Fig).

Similar to previous studies [26–28], the children with overweight/obesity presented an increased percentage of liver fat and total body fat as assessed by magnetic resonance imaging (MRI) as well as obesity-related alterations in metabolic and inflammatory serum parameters such as triglycerides, cholesterol, high-density lipoprotein and low-density lipoprotein (LDL), high sensitive c-reactive protein, alanine aminotransferase (ALAT), adiponectin, leptin, insulin, as well as Homeostatic Model Assessment-Insulin Resistance (HOMA-IR) (S1 Table).

### GHBP and IGF-1 serum levels are increased in children with overweight/obesity independently of age and sex

**Figure 1.**
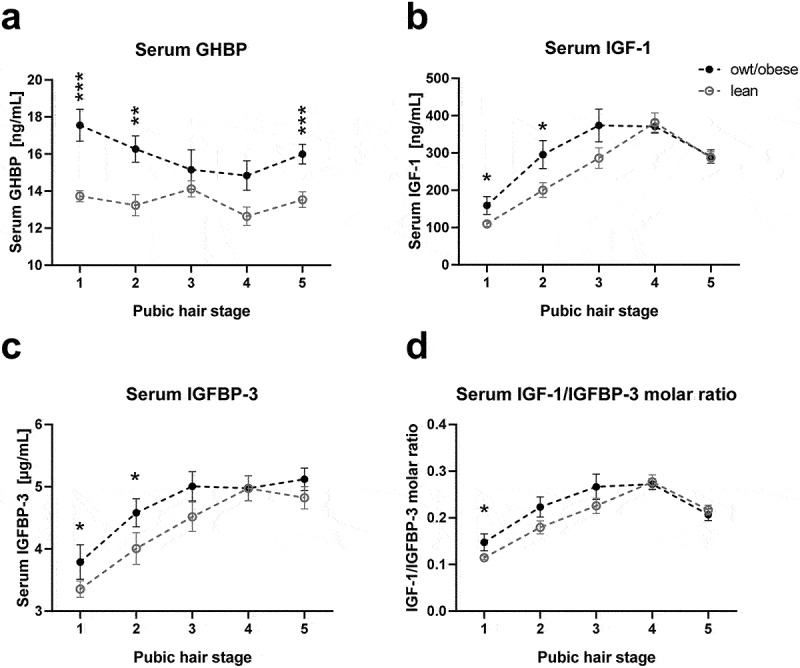
Growth-related serum parameters in lean children and children with overweight/obesity stratified for pubertal stage. Serum levels of (a) growth hormone binding protein (GHBP), (b) insulin-like growth factor-1 (IGF-1), (c) IGF-1 binding protein-3 (IGFBP-3) and (d) the molar ratio of IGF-1 and IGFBP-3 (IGF-1/IGFBP-3) are shown for each pubic hair stage comparing lean children and children with overweight or obesity (owt/obese). Asterisks mark significant differences between the lean and owt/obese group assessed by student’s t-tests (*, p < 0.05; **, p < 0.01; ***, p < 0.001) .

Comparing the mean values of the cohort, children with overweight/obesity were significantly taller compared to lean children and also the serum levels of GHBP, IGF-1 and IGFBP-3 appeared to be increased in children with overweight/obesity (S1 Table). When children were further stratified according to pubertal stage, GHBP serum levels were increased in individuals with overweight/obesity throughout childhood and adolescence (Figure 1(a)), while IGF-1 and IGFBP-3 levels were increased in pre-puberty and early puberty (Figure 1(b,c)) and the IGF-1/IGFBP-3 molar ratio was increased only in pre-pubertal children (Figure 1(d)). However, as children with overweight/obesity were slightly older in our cohort (S1 Table), in particular in pubertal stage 1 (S1 Fig), additional analyses needed to be performed to correct for potential confounders. As IGF-1 and IGFBP-3 levels followed an approximately linear pattern until the age of including 10 years (S2 Fig), only children within this age range were included to the following statistical analyses of IGF-1 and IGFBP-3.

Multiple regression analyses restricted to healthy lean children showed that serum GHBP levels were not associated with age, sex or height standard deviation scores (SDS), while serum IGF-1 and IGFBP-3 and the IGF-1/IGFBP-3 molar ratio were significantly associated with age and/or sex (S3 Table), confirming the relation to physiological development during childhood.

To finally determine the impact of body mass index (BMI) on serum levels independently from age and sex, we performed multiple-regression analyses in the entire cohort, showing that higher BMI SDS was the strongest predictor of increased serum GHBP. For IGF-1 and IGF-1/IGFBP-3 molar ratio, BMI SDS did also independently contribute to variance, although to a minor degree (Table 1). Although this was suggested by Figure 1(c), serum IGFBP-3 was not independently associated with BMI SDS and the differences observed in Figure 1(c) and S1 Table may have resulted from differences in age and sex. Hence, particularly, GHBP is predominantly related to overweight in children.

**Table 1.** Multiple stepwise regression analysis on the effect of BMI SDS on serum levels of GHBP, IGF-1 and IGFBP-3.

Ages included (years)	Dependent variable	Step	Independent variable	*Δ*r^2^	β ± SE	*p*
2–18	**Serum GHBP**	1	BMI SDS	0.133	0.364 ± 0.055	**<0.001**
	(r^2^ = 0.133; *p* < 0.001; n = 285)					
2–10	**Serum IGF-1**	1	Age	0.397	0.500 ± 0.090	**<0.001**
	(r^2^ = 0.509; *p* < 0.001; n = 74)	2	Sex	0.062	−0.269 ± 0.087	**0.003**
		3	BMI SDS	0.051	0.233 ± 0.086	**0.009**
2–10	**Serum IGFBP-3**	1	Age	0.351	0.527 ± 0.095	**<0.001**
	(r^2^ = 0.405; *p* < 0.001; n = 74)	2	Sex	0.054	−0.241 ± 0.095	**0.014**
2–10	**IGF-1/IGFBP-3**	1	Age	0.421	0.459 ± 0.097	**<0.001**
	(r^2^ = 0.432; *p* < 0.001; n = 74)	2	BMI SDS	0.045	0.254 ± 0.093	**0.008**
		3	Sex	0.038	−0.210 ± 0.094	**0.028**

As independent variables age, sex and BMI SDS were included. For IGF-1, IGFBP-3 and IGF-1/IGFBP-3, exclusively children until the age of 10 years were included in the statistical analyses as until this age serum levels followed an approximately linear pattern (S2 Fig). *P*-values <0.05 are highlighted in bold. BMI SDS, body mass index standard deviation score; GHBP, growth hormone binding protein; IGF-1, insulin-like growth factor-1; IGFBP-3, IGF-1 binding protein-3; IGF-1/IGFBP-3, IGF-1 IGFBP-3 molar ratio; Δr^2^, r square change; β ± SE, standardized beta and standard error.

### Association between gene expression in AT cells and serum hormone levels

**Figure 2.**
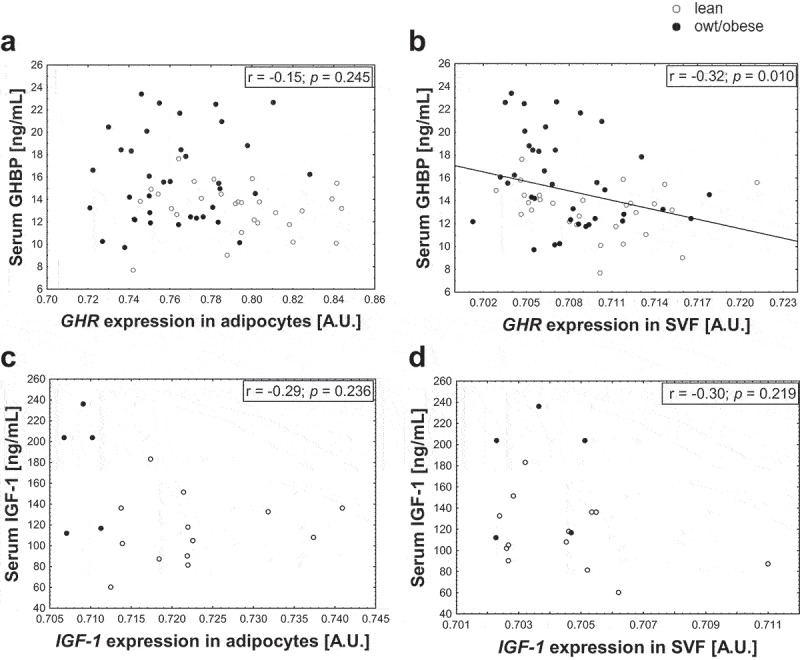
Associations of gene expression of *GHR* and *IGF-1* in adipocytes and SVF cells with serum levels. The relation of serum growth hormone binding protein (GHBP) (shedded from the growth hormone receptor (GHR)) with gene expression of *GHR* in (a) adipocytes and (b) stromal vascular fraction (SVF) cells and the relation of serum insulin-like growth factor-1 (IGF-1) with gene expression of IGF-1 in (c) adipocytes and (d) SVF cells are shown. Regression coefficient r and *p*-value are given and significant correlations are marked with a regression line. For IGF-1 exclusively children until the age of 10 years were included to the analyses as until this age serum levels followed an approximately linear pattern (S2 Fig). For GHBP data from 2–18 years are included. Gene expression data were log_10_-transformed.

Given the association of increased serum GHBP and IGF-1 levels with increasing BMI SDS in children, we assessed if AT cells might contribute to the increase in serum levels observed with overweight/obesity. For this, we assessed whether gene expression levels of *GHR* and *IGF-1* in adipocytes and stromal vascular fraction (SVF) cells were associated with circulating levels in children. There was no positive correlation between gene expression and any of the serum parameters (Figure 2). The only correlations observed were negative associations between *GHR* expression in SVF cells and serum GHBP (Figure 2(b)).

However, it must be considered that children with overweight/obesity have an increased AT mass and a higher number of adipocytes [28] (S1 Table) expressing *GHR* and *IGF-1*. To take this into account, we calculated the total expression from adipocytes per kg body weight per individual. Nonetheless, there again was no correlation with the serum levels (GHBP: r = −0.13, *p* = 0.482; IGF-1: r = −0.22, *p* = 0.590) indicating that, regarding the gene expression, adipocytes may not constitute the major source of elevated circulating GHBP and IGF-1 levels in childhood obesity.

### Association of GHBP serum levels with steatohepatosis

We further observed that in children with overweight/obesity not only the percentage of total body fat (MRI) was increased, but they also already had an increased percentage of liver fat (MRI) (S1 Table). Since the liver is supposed to be the primary source of circulating GHBP, we assessed whether there was an association between the serum levels and liver parameters. Unfortunately, IGF-1 and IGFBP-3 serum levels were not available in the subcohort, in which MRI data were available. Multiple regression analyses revealed that an increased percentage of liver fat is a strong predictor for increased serum GHBP accounting for 35% of serum GHBP variability in this model, while the percentage of total body fat (MRI) did not contribute to the model (Table 2).

**Table 2.** Multiple stepwise regression analyses in order to identify predictors for GHBP serum levels.

Ages Included (years)	Dependent variable	Step	Independent variable	*Δ*r^2^	β ± SE	*p*
2–18	**Serum GHBP**	1	% Liver fat *	0.352	0.580 ± 0.132	**<0.001**
	(r^2^ = 0.436; *p* < 0.001; n = 47)	2	Age	0.051	0.193 ± 0.123	0.124
		3	% Total body fat (MRI)	0.032	0.197 ± 0.126	0.127

As independent variables age, sex, % total body fat (MRI) and % liver fat were included. % liver fat was log_10_-transformed prior to analyses as indicated with an asterisk (*). *P*-values <0.05 are highlighted in bold. GHBP, growth hormone binding protein; Δr^2^, r square change; β ± SE, standardized beta and standard error.

We looked for further correlations with parameters associated with liver function [29] and, indeed, observed positive correlations of serum GHBP with liver transaminases ALAT, aspartate aminotransferase (ASAT), as well as TNF-α and the lipid parameters total cholesterol, LDL cholesterol and triglyceride levels independently from BMI SDS and developmental parameters (Table 3), confirming a potential association of increased serum GHBP with increased liver fat content.

**Table 3.** Association of serum GHBP with metabolic and liver parameters.

Parameter	n	r	*p*
ALAT, µkat/L*	97	0.506	**<0.001**
ASAT, µkat/L*	97	0.460	**<0.001**
Adiponectin, mg/L*	181	−0.033	0.658
Leptin, ng/mL*	189	0.044	0.592
TNF-α, pg/mL*	180	0.181	**0.016**
hsCRP mg/L*	176	0.112	0.125
Cholesterol, mmol/L*	279	0.3391	**<0.001**
LDL cholesterol, mmol/L*	280	0.323	**<0.001**
HDL cholesterol, mmol/L*	297	0.133	0.079
Triglycerides, mmol/L*	93	0.405	**<0.001**
Glucose, mmol/L*	184	−0.124	0.100
Insulin, pmol/L*	181	0.002	0.983
HOMA-IR*	179	0.016	0.839

Analyses were performed using partial correlation with adjustment for age, sex and BMI SDS. *P*-values <0.05 are highlighted in bold. Data of children from age 2–18 years are included. Asterisks (*) indicate that this parameter was log_10_-transformed for statistical analysis. GHBP, growth-hormone binding protein; ALAT, alanine aminotransferase; ASAT, aspartate aminotransferase; hsCRP, high sensitive C-reactive protein; HDL, high-density lipoprotein; LDL, low density lipoprotein; TNF-α, tumour necrosis factor alpha; HOMA-IR, Homeostasis Model Assessment for Insulin Resistance.

### GHR, IGF-1 and IGFBP-3 gene expression is decreased in AT cells in children with overweight/obesity

**Figure 3.**
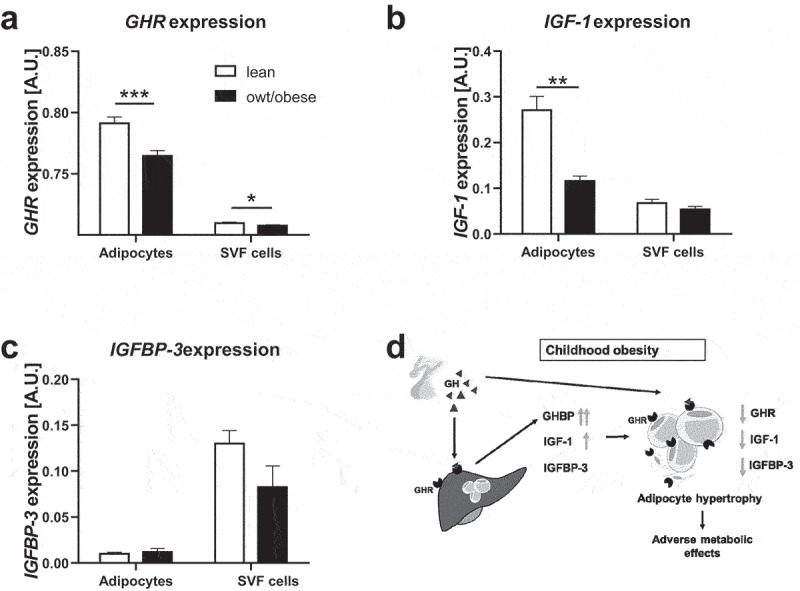
Gene expression of *GHR, IGF-1* and *IGFBP-3* in adipose tissue cells in lean children and children with overweight/obesity. For adipocytes and stromal vascular fraction (SVF) cells gene expression levels of (a) growth hormone receptor (*GHR*), (b) insulin-like growth factor-1 (*IGF-1*) and (c) IGF-1 binding protein-3 (*IGFBP-*3) are shown. For *IGF-1* and *IGFBP-3* expression, data from children between ages 2–10 years are included. (S2 Fig). For *GHR* expression data from 2–18 years are included. Asterisks mark significant differences between lean children and children with overweight/obesity (owt/obese) assessed by student’s t-tests (*, p < 0.05; **, p < 0.01; ***, p < 0.001). For statistical analyses gene expression data were log_10_-transformed. (d) Schematic overview of the proposed role of components of the GH axis in childhood obesity and adipose tissue function.

In order to assess the role of *GHR, IGF-1* and *IGFBP-3* expression in AT, we first compared the gene expression of these factors between mature adipocytes and SVF cells isolated from subcutaneous AT of lean children. *GHR* expression was almost 10-fold increased in adipocytes compared to SVF cells (Figure 3(a)) and *IGF-1* approximately 4-fold higher in adipocytes than in SVF cells (Figure 3(b)), while *IGFBP-3* expression was around 13-fold lower in adipocytes (Figure 3(c)). In line with that, during human adipocyte differentiation of SGBS cells and particularly in late stages of differentiation and subsequent to the *PPARG* increase (S3a Fig.), *GHR* and *IGF-1* expression in differentiated SGBS cells increased >300-fold and >800-fold, respectively, compared to undifferentiated cells (S3b and S3c Fig). In contrast, *IGFBP-3* expression was decreased to 38% in differentiated cells (S3d Fig). Expression of *IGF-1R* (S3e Fig) was not different in differentiated cells, while isoforms A and B of the insulin receptor (*INSR*) (S3f Fig) were around 7- and 5-fold increased, respectively.

Interestingly, gene expression dynamics of *GHR, IGF-1* and *IGFBP-3* was diminished in children with overweight/obesity compared to lean children (Figure 3).

In line with this, after adjusting to the confounding parameters age and sex (S4 Table), gene expression of *GHR* and *IGF-1* in adipocytes as well as *IGFBP-3* expression in SVF cells decreased with increasing BMI SDS (S5 Table).

### GHR, IGF-1 and IGFBP-3 expression in AT may be related to AT function

As children with overweight/obesity showed reduced gene expression of *GHR, IGF-1* and *IGFBP-3* in subcutaneous adipocytes and SVF cells, we next analysed if local expression of these factors in adipocytes and SVF cells was associated with parameters of AT function.

We found that reduced *GHR* expression in adipocytes and *IGF-1* expression in SVF cells was associated with a larger diameter of adipocytes and a reduced number of adipocytes per g AT independently of age, sex and BMI SDS (Table 4), hence indicative for adipocyte hypertrophy. Additionally, reduced *IGFBP-3* expression in adipocytes seemed to be related to a lower number of adipocytes per g AT. Regarding parameters indicative of AT inflammation, we observed an inverse correlation of *IGFBP-3* expression in adipocytes with the number of macrophages and gene expression of *IGFBP-3* in SVF cells was slightly lower in AT samples interspersed with crown-like structures (CLS) than in samples without. We did not find an association of *GHR* or *IGF-1* expression with macrophage infiltration or formation of CLS in AT.

**Table 4.** Association of *GHR, IGF-1* and *IGFBP-3* expression in adipocytes and SVF cells on AT function adjusted for age, sex and BMI SDS.

			*GHR* *		*IGF-1* *		*IGFBP-3* *	
	n		Adipocytes	SVF	Adipocytes	SVF	Adipocytes	SVF
**AT composition**								
Adipocyte diameter, µm	49	r	−0.338	−0.274	−0.136	−0.506	−0.238	−0.214
		*p*	**0.033**	0.087	0.403	**0.001**	0.139	0.185
Number of adipocytes per g ATx10^6^*	49	r	0.364	0.262	0.299	0.402	0.359	0.221
		*p*	**0.021**	0.103	0.061	**0.010**	**0.023**	0.170
**AT inflammation**								
Macrophages per 100 adipocytes*	57	r	−0.128	−0.201	−0.029	−0.049	−0.297	−0.227
		*p*	0.377	0.161	0.842	0.738	**0.036**	0.114
Crown-like structures^b^	32 absent	mean ± SD	1.070 ± 0.416	0.118 ± 0.051	0.216 ± 0.12	0.062 ± 0.03	0.009 ± 0.006	0.103 ± 0.057
	25 present	mean ± SD	0.893 ± 0.487	0.103 ± 0.048	0.168 ± 0.089	0.059 ± 0.022	0.008 ± 0.006	0.069 ± 0.038
		*p*	0.125	0.256	0.105	0.706	0.524	**0.011**
	61	r	−0.075	−0.276	0.035	0.113	−0.093	0.049
Serum hsCRP *		*p*	0.584	**0.040**	0.797	0.406	0.494	0.722
**AT function**								
Basal lipolysis of adipocytes	18	r	0.049	0.136	0.167	−0.126	0.172	0.248
		*p*	0.826	0.629	0.552	0.654	0.540	0.373
Stimulated lipolysis of adipocytes*	19	r	0.426	0.201	0.441	−0.005	0.424	0.390
		*p*	0.100	0.456	0.087	0.985	0.101	0.135
Doubling time of cells, hours*	33	r	−0.330	−0.250	0.105	0.124	−0.135	−0.041
		*p*	0.100	0.218	0.611	0.547	0.510	0.844
Differentiation of SVF cells, %	30	r	−0.075	0.132	0.021	−0.085	−0.123	0.183
		*p*	0.721	0.529	0.922	0.685	0.560	0.382
**Serum parameters**								
Adiponectin, mg/L*	64	r	0.119	0.046	0.109	0.035	0.148	0.177
		*p*	0.370	0.728	0.413	0.790	0.264	0.181
Leptin, ng/mL*	63	r	−0.051	−0.222	−0.003	0.171	0.134	−0.100
		*p*	0.706	0.094	0.981	0.201	0.316	0.453
Glucose, mmol/L*	66	r	−0.078	−0.053	0.154	−0.047	−0.138	−0.224
		*p*	0.546	0.681	0.228	0.712	0.281	0.077
Insulin, pmol/L*	64	r	−0.174	−0.306	0.053	−0.318	−0.093	−0.265
		*p*	0.179	**0.017**	0.686	**0.012**	0.476	**0.039**

Asterisks (*) indicate that this parameter has been log_10_-transformed for statistical analysis. *P*-values <0.05 are highlighted in bold. Data from children age 2–18 years were analysed using partial regression analyses. Gene expression of crown-like structure positive and negative tissues was compared using a student’s test (^b^). BMI SDS, body mass index standard deviation score; GHR, growth hormone receptor; IGF-1, insulin-like growth factor-1; IGFBP-3, IGF-1 binding protein; AT, adipose tissue; SVF, stromal vascular fraction; hsCRP, high sensitive C-reactive protein.

There was no BMI-independent association of *GHR, IGF-1* and *IGFBP-*3 expression in AT cells with basal or isoproterenol-stimulated lipolysis of adipocytes or with the proliferative and adipogenic potential of SVF cells *in vitro*. Also, we did not observe associations with serum adiponectin, leptin or fasting glucose levels, but *GHR, IGF-1* and *IGFBP-*3 expression in SVF cells seemed to be inversely correlated with serum insulin.

## Discussion

We found that circulating GHBP, and in prepubertal children also IGF-1 but not IGFBP-3 are increased in children with overweight/obesity independently from age and sex. We could not confirm our hypothesis that AT expression of these factors is relevantly contributing to the increased serum levels, but found a strong relation of serum GHBP to the amount of liver fat. We found that gene expression of *GHR, IGF-1* and *IGFBP-3* was compromised in AT of children with overweight/obesity and was associated with parameters of adipocyte function such as adipocyte hypertrophy and increased fasting insulin levels (Figure 3(d)).

Although previous and our studies are showing that circulating levels of GHBP are increased in children with obesity [30–32] and that IGF-1 serum levels are increased in children with overweight/obesity during pre-puberty and/or early puberty [2,30,33–36], it is not clear whether expression of *IGF-1* or *GHR/GHBP* in AT directly contributes to these alterations in serum levels. We addressed this question by performing an association study using a unique cohort of children with AT samples and characterization of AT biology and function in addition to anthropometric and metabolic parameters available [28].

While it is known that *GHR* [17] and *IGF-1* [37] are expressed in whole AT in humans, in this study, we further discriminated between freshly isolated human adipocytes and SVF cells from children and showed higher expression of *GHR* and *IGF-1* in adipocytes compared to SVF cells. We did not observe a positive correlation between gene expression in adipocytes and serum parameters, indicating that AT might not be the major source of circulating GHBP and IGF-1 contributing to the overweight-related elevations. In addition, we considered the increased number of adipocytes in individuals with overweight and included the parameters total adipocyte number and total body weight in our analyses and did not see an association. To our knowledge, these relations have not been investigated previously in children, although in children the GH-IGF-1 axis is most important and most dynamic. In particular, IGF-1 serum levels are highly age-dependent and exhibit non-linear patterns over age. Due to high sample sizes in this study, it was possible to investigate a subset of children for which the values run linear, and hence statistical analyses could be performed.

Interestingly, a study investigating the relation between *GHR* expression in intra-abdominal whole AT and serum levels in healthy, adult females also did not find a positive but a negative correlation with circulating GHBP [38]. Our data showing a negative association of serum GHBP with *GHR* expression in SVF cells but not in adipocytes suggest a cell-type-specific relation. It is important to mention that based on our analyses we cannot entirely exclude that GHBP and IGF-1 released from AT might contribute to obesity-related elevations in the serum levels, as the amount of protein released from cells is not necessarily reflected by its gene expression. Wabitsch *et al*. showed that gene expression of *IGF-1* was not altered in differentiated SGBS cells compared to undifferentiated cells, but that secretion of the protein was enhanced with adipogenic differentiation [37]. Also, the process of GHBP shedding from GHR is complex and might depend on the abundance of different isoforms within a cell type as the truncated isoform GHR_1-279_ was suggested to have a higher shedding rate [39].

However, our findings are supported by studies in adipocyte-specific knockout mice showing that a lack of IGF-1 in adipocytes did not alter plasma IGF-1 concentration [19,40], while *Igf-1* expression in the liver was slightly increased. Vice versa, in liver-specific *Igf-1* knockout mice there was a trend towards an up-regulation of *Igf-1* expression in other tissues such as fat tissue [21], suggesting a compensatory feedback mechanism between tissues in order to keep circulating levels constant. Despite there being two adipocyte-specific *Ghr* knock out mouse models [41,42], data regarding circulating GHBP levels have not been reported. Hence, overall this strengthens the hypothesis that AT itself is not a relevant contributor to obesity-related elevations of circulating GHBP and IGF-1.

This implies that probably the liver as the major source of the growth factors and their binding proteins [13–16] may contribute to the obesity-related elevation of serum levels, particularly as it is affected by increased lipid load in obesity itself. Indeed, we found serum levels of GHBP highly associated with the percentage of liver fat in children, while the total body fat was not as relevant. Accordingly, alterations in liver function tests (ALAT) and dyslipidemia were related to elevated GHBP serum levels. Although it is known that there is an association of increased GHBP levels with non-alcoholic fatty liver disease in adults [12], with this study, we for the first time show the relation of serum GHBP with liver fat content already in children. This also suggests that for GHBP the liver rather than the AT may be the major source contributing to elevated serum levels with obesity.

Nevertheless, components of the GH axis are also supposed to play a role in AT function. The composition of AT and its metabolic and secretory function is disturbed early in childhood obesity, likely contributing to cardiometabolic sequelae [28]. We observed that gene expression of *GHR* and *IGF-1* in adipocytes and expression of *IGFBP-3* in SVF cells was decreased in children with overweight/obesity compared to lean children. This follows previous findings in mice and in adult humans for *IGF-1* [19,43] and *GHR* [44] but has not been described in children before, and the previous studies in humans did not discriminate between adipocytes and SVF cells. *IGFBP-3* expression in AT has been previously shown not to be different [43] or increased after weight loss [45]. Compromised gene expression of the factors in AT might affect AT function and promote the development of metabolic disease.

Indeed, we found that low *GHR* expression in adipocytes was associated with a larger adipocyte diameter. This is in line with observations in adipocyte-specific *Ghr* knockout mice that had an increase in white AT mass and adipocyte size [41,42]. In numerous studies, a role of GHR in lipolysis has been confirmed [23,46] and a lack of GHR might result in adipocyte hypertrophy. GH stimulates lipolysis in adipocytes in a GHR-dependent manner [47] via inhibition of lipoprotein lipase and stimulation of the hormone-sensitive lipase [48]. In our study, *GHR* expression in adipocytes did not significantly correlate with the lipolytic rate, which, however, might be due to the relatively small sample size.

Also, regarding IGF-1, we found that reduced expression in SVF cells was associated with increased adipocyte size. A potential explanation would be that proliferation of preadipocytes in AT is impaired with reduced local IGF-1 resulting in fewer and larger adipocytes [24,25]. In the Berlin Fat Mouse, an adipocyte-specific *Igf-1* knockout resulted in a reduction of fat mass. However, the authors did not look in more detail into the AT composition in order to dissect effects on adipocyte hypertrophy or hyperplasia [40]. Effects of IGF-1 on subcutaneous adipocytes are rather mediated by INSR and not by the IGF-1R as *Insr* knockout mice had a 95% decrease in white AT, while *Igf-1r* knockout mice only had a 25% decrease [49]. Accordingly, we and others [50] found gene expression of *INSR*, but not *IGF-1R*, up-regulated during adipogenic differentiation.

As a modulator of bioavailability of IGF-1, IGFBP-3 is known to affect AT function via IGF-1-dependent mechanisms, but also IGF-1-independent mechanisms inhibiting adipocyte differentiation have been suggested [51,52]. This is in line with our data showing that IGFBP-3 is rather down-regulated during differentiation of SGBS cells. We furthermore found a relation of reduced *IGFBP-3* expression in AT cells with obesity and with increased AT inflammation, which fits to previously proposed anti-inflammatory and pro-apoptotic effects [53].

A BMI-independent and inverse association of gene expression of *GHR, IGF-1* and *IGFBP-3* in SVF cells with fasting insulin levels indicates that reduced gene expression might not only be related to AT dysfunction but also to the metabolic state in children.

Thus, our data imply that components of the GH axis might be involved in AT development and function in children and that a local obesity-related decrease of *GHR, IGF-1* and *IGFBP-3* expression in AT might contribute to AT dysfunction such as impaired adipogenic differentiation and adipocyte hypertrophy and may lead to a deterioration of the metabolic state. To the best of our knowledge, there is no other study analysing associations between *GHR, IGF-1* and *IGFBP-3* gene expression in isolated adipocytes and SVF cells with such a comprehensive set of data on AT function and inflammatory and metabolic parameters, neither in adults, nor in children.

A limitation of the study is that the GH status could not be assessed from circulating levels due to its nocturnal pulsatile secretion. In addition, serum levels of IGF-1, IGFBP-3 and GHBP may be partially dependent on further factors, such as physical activity [54] and nutrition intake [55], which may affect the analyses. However, the comparably high sample sizes analysed in our study may compensate for a potential bias due to variations in these factors.

Further, it is important to highlight again that we exclusively looked at gene expression in AT and that the secretion of the proteins might deviate.

The strength of our study is that we analysed the role of growth-related factors in children and not in adults, as here the regulation of growth is most important and dynamic. Furthermore, children present early stages of disease progression and are mostly free of medication and comorbidities, potentially confounding the analyses. In addition, in contrast to many studies investigating obesity-related alterations in AT, we did not only look at human whole AT but also differentiated between adipocytes and SVF cells, which might provide a more detailed insight into processes in AT.

In conclusion, AT may not be the major source for the obesity-related elevations in serum GHBP and IGF-1 in children. However, reduced gene expression of the here investigated components of the GH-axis in AT cells in children with overweight/obesity might contribute to a compromised AT function, potentially further promoting the development of metabolic disease in the state of obesity. Further studies are needed to decipher the specific role of GHR, IGF-1 and IGFBP-3 in AT function and the contribution of AT to serum levels. Finally, in clinics, assessment of growth and growth hormone axis components in children should also take into account obesity and metabolic health.

## Materials and methods

### Study cohort

Our study encompassed 306 children aged 2–18 years from the Leipzig AT Childhood Cohort [28,56] and the Leipzig Atherobesity Childhood Cohort [26]. For the Leipzig AT Childhood Cohort parameters for AT and serum GHBP, IGF-1 and IGFBP-3 levels and metabolic parameters and for the Leipzig Atherobesity Childhood Cohort serum GHBP, MRI data of body fat and liver fat and metabolic parameters were available. Participants were recruited at the University Hospital for Children & Adolescents, Leipzig, Germany, between the years 2009–2015. We included boys and girls with a BMI SDS higher than −1.88 and a height SDS between −2.5 and 3.5. They were free of diseases (diabetes, generalized inflammation, genetic syndromes or permanent immobility), and children from the Leipzig AT Childhood Cohort were additionally free of medication potentially influencing AT biology. Subcutaneous AT samples were obtained from children undergoing elective orthopaedic surgery, herniotomy/orchidopexy or other surgeries as described before [28].

### Anthropometric measurements and laboratory analyses

Height was measured to the nearest of 1 millimetre using a rigid stadiometer. Weight was measured in light underwear to the nearest 0.1 kg using a calibrated scale. Height and BMI values were transformed to sex- and age-specific national reference data and are given in SDS [57]. Pubertal stage was assessed by pubic hair stage according to Tanner [58,59]. Children were classified into weight categories: lean (BMI-SDS between −1.88 and 1.28) and with overweight and obesity (BMI-SDS >1.28 and 1.88), respectively.

The percentage of liver fat and total body fat (MRI) was measured with localized ^1^H magnetic resonance spectroscopy and analysed as previously described [60]. The percentage of liver fat is presented as the mean between the right upper (one measurement) and the right lower (mean from two measurements at the same location) lobe. The percentage of total body fat (MRI) is the sum of the percentage of subcutaneous and visceral AT.

Blood samples were obtained after an overnight fast, immediately centrifuged and stored at −80°C. Serum levels were measured by standard laboratory procedures in the Institute of Laboratory Medicine, Clinical Chemistry and Molecular Diagnostics of the University of Leipzig, Germany, as described in Table S6. HOMA-IR was calculated as HOMA-IR = (Insulin [mU/L]×Glucose [mmol/L])/22.5 [61].

### Isolation of adipocytes and SVF cells and assessment of AT function

Subcutaneous AT samples were processed directly after surgery, and adipocytes and SVF cells were isolated as previously described [28]. Briefly, separated adipocytes were frozen for RNA analyses or fixed in osmium tetroxide for analyses of adipocyte number and size distribution using the Coulter counter (Multisizer III; Beckmann Coulter, Krefeld, Germany). Lipolytic capacity of isolated adipocytes was assessed using the Free Glycerol Reagent (Sigma) and normalized to adipocyte number determined using the Coulter counter method. Lipolytic rate is given as the release of glycerol in ng/mL per 1,000 adipocytes. Isolated SVF cells were frozen for RNA analyses and proliferation and differentiation assay were performed. Furthermore, histological analyses and calculation of AT mass in kg and adipocyte number per gram AT were performed. Detailed protocols for that are described by Landgraf *et al*. [28].

The total *GHR* and *IGF-1* gene expression in adipocytes per kg body weight was calculated by multiplying the normalized gene expression levels with the total number of adipocytes (calculated by multiplying the number of adipocytes per kg AT with the total amount of AT mass in kg) and divided by the total body weight.

### Gene expression analysis

RNA was isolated from the cells, reverse-transcribed and RNA expression was analysed using TaqMan quantitative real-time polymerase-chain reaction using the standard curve method as described previously [62]. Results were normalized using the housekeeper genes TATA-box binding protein, beta-actin and hypoxanthine-guanine phosphoribosyltransferase. All primer and probe sequences or TaqMan assays (Applied Bioscience) are listed in S7 Table.

### Statistical analyses

When not normally distributed, data have been log_10_-transformed for statistical analyses. Multiple regression analyses were performed using a stepwise forward model stopping at a *p*-value of 0.05. The most significant variable at each step was determined according to the smallest *p*-value. Lean and overweight/obese groups were compared using unpaired student’s *t*-test (two-sided) or Chi^2^-test. *P*-values <0.05 were considered statistically significant. Statistical analyses were performed with Statistica 13 software (Dell, Round Rock, US) and GraphPad Prism 6 (GraphPad Software, San Diego, CA).

## Supplementary Material

Supplemental Material

## Data Availability

The datasets used and/or analysed during the current study are available from the corresponding author on reasonable request.
