# Supplementary material for: Associations of GHR, IGF-1 and IGFBP-3 expression in adipose tissue cells with obesity-related alterations in corresponding circulating levels and adipose tissue function in children
Source: Adipocyte. 2022 Nov 22;11(1):630–42. doi: 10.1080/21623945.2022.2148886 (PMC9683049; doi:10.1080/21623945.2022.2148886)
Supplement: Supplemental Material [file KADI_A_2148886_SM1747.docx]

**Supporting information**

**S1 Table. Characteristics of the cohort (N=306) and comparison of different parameters between lean children and children with overweight/obesity.**

|  |  |  | **Lean** |  | **Overweight/obese** | | | | | | |  |
| --- | --- | --- | --- | --- | --- | --- | --- | --- | --- | --- | --- | --- |
| **Anthropometry** | **n** | **mean** | **SD** | **range** | **n** | | **mean** | | **SD** | | **range** | ***p*** |
| Sex | 157 | female: 75 (47.8 %),  male: 82 (52.2 %) | | | 149 | female: 74 (48.7 %),  male: 75 (51.3 %) | | | | | | 0.740^a^ |
| Age | 157 | 11.05 | 4.80 | 2 - 18.72 | 149 | | 12.89 | | 3.63 | | 2.35 - 18.71 | **<0.001** |
| Height SDS | 157 | 0.03 | 1.10 | -2.47 - 3.07 | 149 | | 0.65 | | 1.08 | | -2.23 - 3.3 | **<0.001** |
| BMI SDS | 157 | -0.16 | 0.82 | -1.8 - 1.24 | 149 | | 2.37 | | 0.63 | | 1.32 - 4.34 | **<0.001** |
| Body weight, kg | 157 | 40.42 | 18.43 | 9 - 82.45 | 149 | | 77.08 | | 24.46 | | 14 - 137.6 | **<0.001** |
| Pubic hair stage | 139 | 2.75 | 1.69 | 1 - 5 | 139 | | 3.35 | | 1.57 | | 1 - 5 | **0.001^a^** |
| Liver fat, % (MRI) | 24 | 1.20 | 0.64 | 0.1 - 2.45 | 28 | 9.63 | | 12.32 | | 0.3 - 47.78 | | **<0.001*** |
| Total body fat, % (MRI) | 24 | 20.35 | 11.09 | 7.36 - 58.64 | 27 | | 34.34 | | 8.13 | | 13.42 - 46.21 | **<0.001** |
| AT mass, kg | 81 | 10.02 | 6.71 | 1.94 - 31.25 | 66 | | 26.61 | | 10.86 | | 4.16 - 60 | **<0.001*** |
| Total number of adipocytes x10^9^ | 20 | 23.16 | 14.78 | 8.12 - 58.45 | 30 | | 50.61 | | 20.18 | | 22.13 - 107.14 | **<0.001*** |
| Total number of AT-derived SVF cells x10^8^ | 18 | 13.10 | 11.20 | 1.83 - 49.46 | 21 | | 31.49 | | 21.78 | | 7.99 - 95.11 | **<0.001*** |
| **Serum parameters** |  |  |  |  |  | |  | |  | |  |  |
| **GH axis** |  |  |  |  |  | |  | |  | |  |  |
| GHBP, ng/mL | 145 | 13.55 | 2.21 | 7.66 - 18.92 | 140 | | 16.01 | | 3.83 | | 8.9 - 28.18 | **<0.001** |
| IGF-1, ng/mL | 107 | 198.87 | 117.51 | 32.3 - 484.6 | 81 | | 284.19 | | 124.94 | | 38.5 – 549.4 | **<0.001** |
| IGFBP-3, µg/mL | 107 | 3.97 | 1.07 | 1.47 - 6.12 | 81 | | 4.67 | | 1.01 | | 1.96 - 6.96 | **<0.001** |
| IGF-1/IGFBP3 molar ratio | 107 | 0.17 | 0.07 | 0.06 - 0.36 | 81 | | 0.21 | | 0.08 | | 0.07 - 0.37 | **<0.001** |
| **Metabolic factors** |  |  |  |  |  | |  | |  | |  |  |
| Triglycerides, mmol/L | 37 | 0.91 | 0.47 | 0.43 - 2.25 | 56 | | 1.22 | | 0.68 | | 0.5 - 3.74 | **0.015*** |
| Cholesterol, mmol/L | 144 | 3.88 | 0.72 | 2.46 - 6.45 | 141 | | 4.00 | | 0.80 | | 2.25 - 6.27 | 0.240* |
| HDL cholesterol, mmol/L | 144 | 1.35 | 0.32 | 0.71 - 2.44 | 141 | | 1.15 | | 0.27 | | 0.47 - 2.02 | **<0.001*** |
| LDL cholesterol, mmol/L | 144 | 2.18 | 0.55 | 1 - 3.8 | 142 | | 2.35 | | 0.65 | | 0.95 - 4.1 | **0.016*** |
| hsCRP, mg/L | 106 | 0.69 | 0.75 | 0.08 - 4.5 | 75 | | 1.22 | | 0.99 | | 0.08 – 4.5 | **<0.001*** |
| TNF-α, pg/mL | 104 | 2.08 | 0.91 | 0.52 - 6.02 | 79 | | 1.98 | | 0.76 | | 0.6 - 4.78 | 0.511* |
| ALAT, µkat/L | 38 | 0.26 | 0.09 | 0.07 - 0.52 | 59 | | 0.47 | | 0.35 | | 0.13 - 1.77 | **<0.001*** |
| ASAT, µkat/L | 38 | 0.43 | 0.10 | 0.24 - 0.73 | 59 | | 0.46 | | 0.16 | | 0.19 - 0.98 | 0.233* |
| Adiponectin, mg/L | 106 | 8.61 | 4.41 | 2.1 - 25.6 | 81 | | 6.10 | | 3.07 | | 1.7 - 15.9 | **<0.001*** |
| Leptin, ng/mL | 109 | 6.06 | 5.25 | 0.2 – 24.1 | 84 | | 28.38 | | 22.11 | | 0.6 - 99 | **<0.001*** |
| Glucose, mmol/L | 107 | 4.57 | 0.53 | 2.47 - 6.06 | 83 | | 4.66 | | 0.62 | | 3.14 - 6.23 | 0.280 |
| Insulin, pmol/L | 103 | 43.16 | 32.89 | 4.1 - 159.6 | 79 | | 108.28 | | 70.66 | | 5 - 309.8 | **<0.001*** |
| HOMA-IR | 103 | 1.26 | 1.04 | 0.13 - 5.64 | 79 | | 3.22 | | 2.28 | | 0.14 - 10.12 | **<0.001*** |

Differences within each parameter between lean children and children with overweight/obesity have been analysed using student’s t-test. Categorical parameters have been compared using the Chi²-Test as indicated with ^a^. A parameter has been log_10_-transformed for analyses if the *p*-value is marked with an asterisk (*). *P*-values <0.05 are highlighted in bold. SD, standard deviation; SDS, SD score; AT, adipose tissue; SVF, stromal vascular fraction; GHBP, growth hormone binding protein; IGF-1, insulin-like growth factor-1; IGFBP-3, IGF-1 binding protein-3; HDL, high-density lipoprotein; LDL, low density lipoprotein; hsCRP, high sensitive C-reactive protein; TNF-α, tumor necrosis factor alpha; ALAT, alanine transaminase; ASAT, aspartate transaminase; HOMA-IR, Homeostasis Model Assessment for Insulin Resistance.

**S2 Table. Comparison of different parameters regarding the subcutaneous AT between lean children and children with overweight/obesity**

|  |  |  | **Lean** |  | **Overweight/obese** | | | |  |
| --- | --- | --- | --- | --- | --- | --- | --- | --- | --- |
| **Gene expression levels, [A.U.]** | **n** | **mean** | **SD** | **range** | **n** | **mean** | **SD** | **range** | ***p*** |
| Adipocytes *GHR* | 31 | 1.20 | 0.42 | 0.52 - 1.98 | 40 | 0.83 | 0.37 | 0.26 - 1.89 | **<0.001*** |
| Adipocytes *IGF-1* | 31 | 0.25 | 0.11 | 0.07 - 0.51 | 40 | 0.16 | 0.08 | 0.05 - 0.39 | **<0.001*** |
| Adipocytes *IGFBP-3* | 31 | 0.01 | 0.01 | 0 - 0.02 | 40 | 0.01 | 0.01 | 0 - 0.02 | 0.375* |
| SVF *GHR* | 31 | 0.13 | 0.05 | 0.05 - 0.26 | 40 | 0.10 | 0.04 | 0.03 - 0.22 | **0.036*** |
| SVF *IGF-1* | 31 | 0.06 | 0.02 | 0.03 - 0.14 | 40 | 0.06 | 0.03 | 0.03 - 0.16 | 0.390* |
| SVF *IGFBP-3* | 31 | 0.11 | 0.05 | 0.02 - 0.25 | 40 | 0.07 | 0.04 | 0.02 - 0.22 | **<0.001*** |
| Total adipocyte  *GHR* expression *10^9^ per kg BW | 15 | 0.64 | 0.39 | 0.26 - 1.45 | 22 | 0.59 | 0.51 | 0.13 - 1.85 | 0.712* |
| Total adipocyte *IGF-1* expression x10^9^per kg BW | 15 | 0.12 | 0.09 | 0.03 - 0.4 | 22 | 0.11 | 0.07 | 0.03 - 0.35 | 0.649* |
| **AT function** |  |  |  |  |  |  |  |  |  |
| Adipocyte diameter , µm | 20 | 114.24 | 12.01 | 90.91 - 131.2 | 29 | 127.14 | 13.43 | 98.01 - 146.2 | **0.001** |
| Number of adipocytes per g ATx10^6^ | 20 | 2.19 | 0.62 | 1.20 - 3.69 | 29 | 1.82 | 0.52 | 1.00 - 3.01 | **0.031*** |
| Macrophages per 100 adipocytes | 25 | 12.12 | 8.97 | 0 - 29 | 32 | 22.59 | 22.22 | 0 - 115 | **0.032*** |
| Crown-like structures | 25 | absent: 20 (80.0%),  present: 5 (20.0 %) | | | 32 | absent: 12 (37.5 %),  present: 20 (62.5 %) | | | **0.001^a^** |
| Basal lipolysis of adipocytes | 7 | 0.47 | 0.19 | 0.2 - 0.71 | 11 | 0.38 | 0.21 | 0.18 - 0.74 | 0.351 |
| Stimulated lipolysis of adipocytes | 7 | 2.07 | 0.98 | 0.6 - 3.77 | 12 | 2.23 | 1.32 | 0.99 - 5.08 | 0.819* |
| Doubling time of cells, hours | 13 | 146.20 | 97.44 | 30.8 - 366.79 | 20 | 110.77 | 91.68 | 17.8 - 303.6 | 0.181* |
| Differentiation of SVF cells, % | 11 | 23.85 | 16.29 | 4 - 46.62 | 19 | 21.76 | 14.15 | 0.24 - 57.65 | 0.713 |

Differences within each parameter between lean children and children with overweight/obesity have been analysed using student’s t-test. Categorical parameters have been compared using the Chi²-Test as indicated with ^a^. A parameter has been log_10_-transformed for analyses if the *p*-value is marked with an asterisk (*). *P*-values <0.05 are highlighted in bold. SD, standard deviation; SDS, SD score; AT, adipose tissue; GHR, growth hormone receptor; IGF-1, insulin-like growth factor-1; IGFBP-3, IGF-1 binding protein-3; SVF, stromal vascular fraction; BW, body weight.

**S1 Fig. Height SDS and age in lean children and children with overweight/obesity stratified for pubertal stage**

Height standard deviation scores (SDS) and age in years are shown for lean children and children with overweight/obesity (owt/obese) for each pubic hair stage. Asterisks mark significant differences between the lean and owt/obese group assessed by student’s t-tests (*, *p*<0.05; **, *p*<0.01).

**
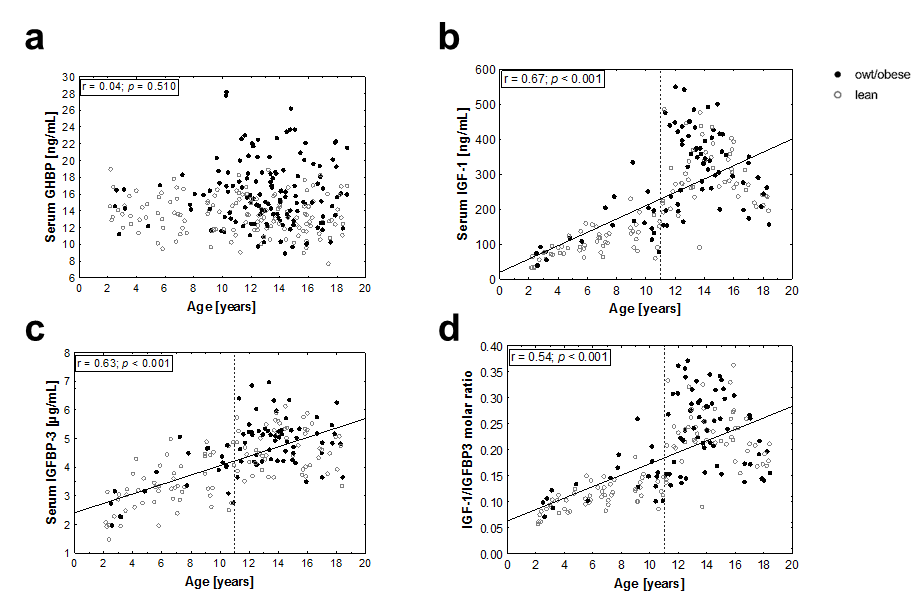
**

**S2 Fig. Serum levels of lean children and children with overweight/obesity across the age**

(a) Growth hormone binding protein (GHBP), (b) insulin-like growth factor 1 (IGF-1), (c) IGF-binding protein-3 (IGFBP-3) and (d) the molar ratio of IGF-1 and IGFBP-3 (IGF-1/IGFBP-3) are shown. Regression coefficient r and *p*-value are given and significant correlations with age are marked with a regression line (solid line). For b-d, serum levels showed an approximately linear increase until the age of 10.999 years (identified by visual inspection) and the age cut-off is indicated by dotted lines.

**S3 Table. Multiple stepwise regression analyses on the impact of sex, age and height SDS on serum GHBP, IGF-1 and IGFBP-3 in lean children**.

| **Ages included**  **(years)** | **Dependent variable** | **Step** | **Independent variable** | **Δr²** | **β ± SE** | ***p*** |
| --- | --- | --- | --- | --- | --- | --- |
| 2-18 | **Serum GHBP** | 1 | Age | 0.015 | -0.121 ± 0.083 | 0.146 |
|  | (r²=0.015; *p*=0.146; n=145) |  |  |  |  |  |
| 2-10 | **Serum IGF-1** | 1 | Age | 0.442 | 0.662 ± 0.105 | **<0.001** |
|  | (r²=0.480; *p*<0.001; n=54) | 2 | Sex | 0.024 | -0.161 ± 0.105 | 0.131 |
|  |  | 3 | Height SDS | 0.014 | 0.117 ±0.102 | 0.255 |
| 2-10 | **Serum IGFBP-3** | 1 | Age | 0.302 | 0.488 ± 0.115 | **<0.001** |
|  | (r²=0.373; *p*<0.001, n=54) | 2 | Sex | 0.56 | -0.244 ± 0.115 | **0.039** |
|  |  | 3 | Height SDS | 0.015 | 0.121 ± 0.112 | 0.287 |
| 2-10 | **IGF-1/IGFBP-3** | 1 | Age | 0.382 | 0.618 ± 0.109 | **<0.001** |
|  | (r²=0.382; *p*<0.001, n=54) |  |  |  |  |  |

As independent variables age, sex and height SDS were included. For IGF-1, IGFBP-3 and IGF-1/IGFBP-3, exclusively serum levels of children until the age of 10 years were included to the statistical analyses as until this age serum levels followed an approximately linear pattern (S2 Fig). *P*-values <0.05 are highlighted in bold. SDS, standard deviation score; GHBP, growth hormone binding protein; IGF-1, insulin-like growth factor-1; IGFBP-3, IGF-1 binding protein-3; IGF-1/IGFBP-3, IGF-1 IGFBP-3 molar ratio; Δr², r square change; β ± SE, standardized beta and standard error.

**S3 Fig. Gene expression of components of the GH axis during adipocyte differentiation *in vitro* in SGBS cells**

Human pre-adipocytes derived from an infant with Simpson–Golabi–Behmel syndrome (SGBS) were kindly provided by M. Wabitsch (Division of Pediatric Endocrinology and Diabetes, University of Ulm, Ulm, Germany) [[1](#_ENREF_1)]. Cells were cultured and differentiated for 12 days as previously described [[2](#_ENREF_2)]. Gene expression of (a) peroxisome proliferator-activated receptor gamma (*PPARG)*, (b) growth hormone receptor (*GHR),* (c) insulin-like growth factor-1 (*IGF-1)*, (d) IGF-1 binding protein-3 (*IGFBP-3),* (e*)* IGF-1 receptor *(IGF-1R*) and (f) insulin receptor isoforms A (*INSR-A*) and B (*INSR-B*) in Simpson-Golabi-Behmel syndrome cells during adipocyte differentiation. Results are given as mean fold change ± SEM compared to day 0 (dashed line) of differentiation (n=4). Experiments were measured in three technical replicates.

**S4 Table. Multiple stepwise regression analyses on the impact of sex, age and height SDS on gene expression levels of *GHR*, *IGF-1* and *IGFBP-3* in AT cells in lean children (n=31)**.

|  | **Dependent variable** | **Step** | **Independent variable** | **Δr²** | **β ± SE** | ***p*** |
| --- | --- | --- | --- | --- | --- | --- |
|  | **Adipocytes** | 1 | Sex | 0.132 | 0.363 ± 0.173 | **0.044** |
| ***GHR*** | (r²=0.132; *p*=0.044) |  |  |  |  |  |
|  | **SVF** | 1 | Age | 0.178 | -0.444 ± 0.166 | **0.013** |
|  | (r²=0.285; *p*=0.026) | 2 | Height SDS | 0.067 | 0.280 ± 0.164 | 0.099 |
|  |  | 3 | Sex | 0.041 | -0.208 ± 0.167 | 0.222 |
|  | **Adipocytes** | 1 | Height SDS | 0.113 | -0.336 ± 0.175 | 0.065 |
| ***IGF-1*** | (r²=0.113; *p*=0.065) |  |  |  |  |  |
|  | **SVF** | 1 | Age | 0.110 | -0.389 ± 0.173 | **0.032** |
|  | (r²=0.194; *p*=0.049) | 2 | Sex | 0.084 | -0.296 ± 0.173 | 0.098 |
|  | **Adipocytes** | 1 | Age | 0.307 | -0.554 ± 0.155 | **0.001** |
| ***IGFBP-3*** | (r²=0.307; *p*=0.001) |  |  |  |  |  |
|  | **SVF** | 1 | Age | 0.211 | -0.519 ± 0.161 | **0.003** |
|  | (r²=0.301; *p*=0.007) | 2 | Sex | 0.090 | -0.305 ± 0.1161 | 0.068 |

As independent variables age, sex and height SDS were included. *P*-values <0.05 are highlighted in bold. In contrast to the serum levels, patterns of gene expression in AT cells across age appeared linear for all three genes (data not shown), hence gene expression data from children at ages 2-18 years were analyzed.

Gene expression data were log_10_-transformed for analyses. GHR, growth hormone receptor; IGF-1, insulin-like growth factor-1; IGFBP-3, IGF-1 binding protein-3; SVF, stromal vascular fraction; Δr², r square change; β ± SE, standardized beta and standard error.

**S5 Table. Correlation between BMI SDS and gene expression levels of *GHR*, *IGF-1* and *IGFBP-3* in adipocytes and SVF cells adjusted for age and sex (n=71).**

| **BMI SDS vs. gene expression of:** | ***GHR*** | | ***IGF-1*** | | ***IGFBP-3*** | |
| --- | --- | --- | --- | --- | --- | --- |
| **In cell types:** | **r** | ***p*** | **r** | ***p*** | **r** | ***p*** |
| **Adipocytes** | -0.431 | **<0.001** | -0.417 | **<0.001** | 0.088 | 0.471 |
| **SVF** | -0.210 | 0.084 | -0.028 | 0.820 | -0.324 | **0.007** |

Children from ages 2-18 have been included for all three genes. Gene expression data have been log_10_-transformed for analyses. As the gene expression levels in part were related to age and/or sex in lean children (**S4 Table**) analyses were adjusted to age and sex. *P*-values <0.05 are highlighted in bold. BMI SDS, body mass index standard deviation score; GHR, growth hormone receptor; IGF-1, insulin-like growth factor-1; IGFBP-3, IGF-1 binding protein-3; SVF, stromal vascular fraction.

**S6 Table. Methods for measurements of serum parameters.**

| Serum parameter | Measurement method | Source |
| --- | --- | --- |
| Growth hormone receptor-binding protein (GHBP) | ELISA | DSL, Sinsheim, Germany |
| Insulin-like growth factor (IGF-1) | automated CLIA iSYS | IDS, Boldon/Tyne & Wear, UK |
| IGF-binding protein 3 (IGFBP-3) | automated CLIA iSYS | IDS, Boldon/Tyne & Wear, UK |
| Adiponectin | ELISA | Mediagnost, Reutlingen, Germany |
| Leptin | ELISA | Mediagnost, Reutlingen, Germany |
| Glucose | Photometric measurement | Cobas Roche, Mannheim, Germany |
| High sensitivity c-reactive protein (hsCRP) | Photometric measurement | Cobas Roche, Mannheim, Germany |
| Total cholesterol | Photometric measurement | Cobas Roche, Mannheim, Germany |
| High density lipoprotein (HDL) | Photometric measurement | Cobas Roche, Mannheim, Germany |
| Low-density lipoprotein (LDL) | Photometric measurement | Cobas Roche, Mannheim, Germany |
| Triglycerides | Photometric measurement | Cobas Roche, Mannheim, Germany |
| Alanine transaminase (ALAT) | Photometric measurement | Cobas Roche, Mannheim, Germany |
| Aspartate aminotransferase (ASAT) | Photometric measurement | Cobas Roche, Mannheim, Germany |
| Insulin | CLIA | Liaison, DiaSorin, Dietzenbach, Germany |
| Tumor necrosis factor alpha (TNF-α) | ELISA | R&D, Minneapolis, Canada |

## ELISA, Enzyme-linked immunosorbent assay; CLIA, chemiluminescence immunoassay

**S7 Table. Primers and probes or TaqMan assays used for quantitative real-time polymerase chain reaction**.

| **Symbol** | **Gene name** | **Forward primer** | **Reverse primer** | **Probe** |
| --- | --- | --- | --- | --- |
| ***ACTB*** | beta-actin | CGAGCGCGGCTACAGCTT | CCTTAATGTCACGCACGATTT | ACCACCACGGCCGAGCGG |
| ***GHR*** | Growth hormone receptor | TTGGAATATTTGGGCTAACAGTGA | CCTCCTCTAATTTTCCTTCCTTGAG | AGGATTAAAATGCTGATTCTGCCCCCAG |
| ***HPRT*** | Hypoxanthine-guanine phosphoribosyltransferase | GGCAGTATAATCCAAAGATGGTCAA | GTCTGGCTTATATCCAACACTTCGT | CAAGCTTGCTGGTGAAAAGGACCCC |
| ***IGF-1*** | Insulin-like growth factor-1 | GCAATGGGAAAAATCAGCAG | GAGGAGGACATGGTGTGCA | CTTCACCTTCAAGAAATCACAAAAGCAGCA |
| ***IGF-1R*** | Insulin-like growth factor-1 receptor | TGCAGCGCCTCCAACTTC | GGTCACTGGCCCAGGAAT | ATCTGCTCCTGCGGGCATAGTCCT |
| ***IGFBP-3*** | Insulin-like growth factor-1 binding protein-3 | TaqMan Assay from ThermoScientific, Applied Bioscience: hs00365742_g1 | |  |
| ***InsR-A*** | Insulin receptor isoform A | TGAGGATTACCTGCACAACG | ACCGTCACATTCCCAACATC | TCCCCAGGCCATCT |
| ***InsR-B*** | Insulin receptor isoform B | CGTCCCCAGAAAAACCTCTTC | GGACCTGCGTTTCCGAGAT | CCGAGGACCCTAGGC |
| ***PPARG*** | Peroxisome proliferator-activated receptor gamma | GATCCAGTGGTTGCAGATTACAA | GAGGGAGTTGGAAGGCTCTTC | TGACCTGAAACTTCAAGAGTACCAAAGTGCAA |
| ***TBP*** | TATA-box binding protein | TTGTAAACTTGACCTAAAGACCATTGC | TTCGTGGCTCTCTTATCCTCATG | AACGCCGAATATAATCCCAAGCGGTTTG |

Forward and reverse primers are given in 5´-3´direction. Probes were labelled with the reporter 5’-FAM or 5’-HEX for *TBP* and the quencher 3’-TAMRA. Primers for *GHR* target the full-length isoform.

**Supporting Information – References**

1. Wabitsch M, Heinze E, Debatin KM, et al. IGF-I- and IGFBP-3-expression in cultured human preadipocytes and adipocytes. Hormone and metabolic research = Hormon- und Stoffwechselforschung = Hormones et metabolisme. 2000 Nov-Dec;32(11-12):555-9.

2. Körner A, Wabitsch M, Seidel B, et al. Adiponectin expression in humans is dependent on differentiation of adipocytes and down-regulated by humoral serum components of high molecular weight. Biochemical and biophysical research communications. 2005 Nov 18;337(2):540-50.
